# Ubiquitin E3 ligase activity of *Ralstonia solanacearum* effector RipAW is not essential for induction of plant defense in *Nicotiana benthamiana*

**DOI:** 10.3389/fmicb.2023.1201444

**Published:** 2023-05-24

**Authors:** Xue Ouyang, Jialan Chen, Zhimao Sun, Rongbo Wang, Xuan Wu, Benjin Li, Congfeng Song, Peiqing Liu, Meixiang Zhang

**Affiliations:** ^1^Department of Plant Pathology, Nanjing Agricultural University, Nanjing, China; ^2^National Engineering Laboratory for Endangered Medicinal Resource Development in Northwest China, Key Laboratory of Medicinal Resources and Natural Pharmaceutical Chemistry of Ministry of Education, College of Life Sciences, Shaanxi Normal University, Xi’an, China; ^3^Fujian Key Laboratory for Monitoring and Integrated Management of Crop Pests, Institute of Plant Protection, Fujian Academy of Agricultural Sciences, Fuzhou, China

**Keywords:** RipAW, *Ralstonia solanacearum*, cell death, plant immunity, SGT1

## Abstract

As one of the most destructive bacterial phytopathogens, *Ralstonia solanacearum* causes substantial annual yield losses of many important crops. Deciphering the functional mechanisms of type III effectors, the crucial factors mediating *R. solanacearum*-plant interactions, will provide a valuable basis for protecting crop plants from *R. solanacearum*. Recently, the NEL (novel E3 ligase) effector RipAW was found to induce cell death on *Nicotiana benthamiana* in a E3 ligase activity-dependent manner. Here, we further deciphered the role of the E3 ligase activity in RipAW-triggered plant immunity. We found that RipAW^C177A^, the E3 ligase mutant of RipAW, could not induce cell death but retained the ability of triggering plant immunity in *N. benthamiana*, indicating that the E3 ligase activity is not essential for RipAW-triggered immunity. By generating truncated mutants of RipAW, we further showed that the N-terminus, NEL domain and C-terminus are all required but not sufficient for RipAW-induced cell death. Furthermore, all truncated mutants of RipAW triggered ETI immune responses in *N. benthamiana*, confirming that the E3 ligase activity is not essential for RipAW-triggered plant immunity. Finally, we demonstrated that RipAW- and RipAW^C177A^-triggered immunity in *N. benthamiana* requires SGT1 (suppressor of G2 allele of *skp1*), but not EDS1 (enhanced disease susceptibility), NRG1 (N requirement gene 1), NRC (NLR required for cell death) proteins or SA (salicylic acid) pathway. Our findings provide a typical case in which the effector-induced cell death can be uncoupled with immune responses, shedding new light on effector-triggered plant immunity. Our data also provide clues for further in-depth study of mechanism underlying RipAW-induced plant immunity.

## Introduction

Plant diseases cause substantial yield losses of crops every year (Savary et al., 2019). Deciphering the molecular mechanisms underlying the plant-pathogen interactions will provide a valuable basis for protecting crops from these pathogens. During plant-pathogen interactions, pathogens usually secrete effectors as powerful weapons of invasion to facilitate their infection. However, after long-term confrontation with pathogens, plants have evolved effector-triggered immunity (ETI) accordingly to recognize pathogens, triggering hypersensitive response (HR) to restrict pathogen invasion (Jones and Dangl, 2006).

ETI is mediated by resistance protein (R protein) which is a kind of highly sensitive immune receptor (Jones and Dangl, 2006; Ngou et al., 2022a,b). Nucleotide-binding domain leucine-rich repeat receptors (NLRs), are the largest class of R proteins, and they are grouped into three categories according to their N-terminal signal transduction domains: Toll/interleukin-1 receptor/resistance protein (TIR) NLRs (TNLs), coiled-coil (CC) NLRs (CNLs) and RPW8-like CC domain (RPW8) NLRs (RNLs) (Jones et al., 2016). SGT1 (suppressor of G2 allele of *skp1*), an essential component of ETI, is required for plant immunity triggered by most NLRs by regulating their stability and activation (Azevedo et al., 2002; Muskett and Parker, 2003; Kadota et al., 2010). Except SGT1, EDS1 (enhanced disease susceptibility) also plays an important role in TNL-mediated immunity (Wiermer et al., 2005; Schultink et al., 2017). RNLs, such as NRG1 (N requirement gene 1) and NRC (NLR required for cell death), do not directly recognize pathogen effector proteins, but usually act downstream of TNLs and CNLs to activate immune signals and induce cell death, respectively (Peart et al., 2005; Wu et al., 2017), so they are called helper NLRs. The major defense hormone salicylic acid (SA) also plays an essential role in regulating plant immunity and programed cell death (Fu et al., 2012) and its signaling is well-integrated into NLR signaling pathways (Zhou and Zhang, 2020).

The bacterial pathogen *Ralstonia solanacearum*, one of the most destructive phytopathogens, can infect more than 200 plant species over a broad geographical range (Genin and Denny, 2012). The extensive genetic diversity of *R. solanacearum* strains causes serious bacterial wilt diseases, leading to great economic losses of many important crops worldwide every year (Qi et al., 2022). *R. solanacearum* secretes more than 70 type III effectors (T3Es) into plant cells as weapon to promote infection (Genin and Denny, 2012). Increasing studies showed that some *R. solanacearum* T3Es are endowed with enzymatic activities which function in its interaction with plants, such as RipTPS with trehalose-phosphate synthase activity (Poueymiro et al., 2014) and RipN with nudix hydrolase activity (Sun et al., 2019). Strikingly, 13 effectors in *R. solanacearum* have ubiquitin ligase structural domains and probably are endowed with ubiquitin ligase activity (Peeters et al., 2013). The presence of such a high abundance of ubiquitin ligases implies that ubiquitination may be an important mechanism by which *R. solanacearum* T3Es function to influence plant immunity.

Ubiquitination is a highly conserved eukaryote-specific post-translational modification of protein and plays an important role in regulating protein function (Rohde et al., 2007). The process of ubiquitin modification is mediated by a ubiquitin-activating enzyme (E1), a limited number of ubiquitin-conjugating enzymes (E2s) and lots of ubiquitin-ligating enzymes (E3s) (Rytkonen and Holden, 2007). E3 ubiquitin ligases are responsible for the specific recognition of target proteins and their subsequent ubiquitination, and therefore are considered as the most important component of ubiquitination (Buetow and Huang, 2016). According to the domains it contains and the way it binds to substrates, E3 ubiquitin ligases are classified into HECT (homologous to the E6-AP carboxyl terminus), U-box, RING (really interesting new gene), CRLs (cullin-RING ligases) and NEL (novel E3 ligase) (Vierstra, 2009). Among these, the NEL effector usually contains a typical LRR motif and cysteines that are essential for its ubiquitin ligase activity (Singer et al., 2013). Many effectors with NEL structural domains have been reported to affect plant immunity. For example, IpaH9.8 in *Shigella dysenteriae* degrades guanylate-binding protein (GBP) in host cells by ubiquitination, suppressing host immune response and hence promoting pathogen invasion (Li et al., 2017). RipV2 in *R. solanacearum* UW551 strain and RipAW in RS1000 strain depend on their NEL activities to inhibit plant PTI response and facilitate *R. solanacearum* infection in hosts (Cheng et al., 2021).

Recently, Niu et al. (2022) demonstrates that RipAW in *R. solanacearum* GMI1000 strain induces cell death in *Nicotiana benthamiana* and this process requires its E3 ligase activity. However, cell death is not always coupled with immune responses (Greenberg et al., 2000; Lapin et al., 2019; Yang et al., 2022). To further decipher the relationship of RipAW-triggered immunity with its NEL activity, we determined plant immunity triggered by RipAW^C177A^ (the E3 ligase mutant) and RipAW’s truncated derivatives including RipAW^N1-90^, RipAW^NEL^, RipAW^C322-448^, RipAW ΔN and RipAW ΔC. We found that the E3 ligase activity was not essential for RipAW-triggered immunity. Furthermore, we explored the mechanism how RipAW triggered plant immunity by defining the roles of important immune regulators such as SGT1, EDS1, NRG1 and NRC in RipAW-triggered immunity. Our data provide basis for further in-depth study of the mechanisms behind RipAW-triggered plant immunity.

## Materials and methods

### Plant growth conditions and bacterial strains

Wild-type *N. benthamiana* and *eds1*, *nrg1* (Qi et al., 2018), *nrc2/3/4* (Kourelis et al., 2022) mutants were grown in a walk-in chamber at 24°C under long-day conditions (16 h light/8 h dark). *Escherichia coli* strain DH5α and *Agrobacterium tumefaciens* strain GV3101 were used for cloning and gene expression, respectively. They were cultured on Luria-Bertani (LB) agar plates or in LB liquid medium with appropriate antibiotics at 37°C and 28°C, respectively. *R. solanacearum* strain CQPS-1 (Liu et al., 2017) was cultured at 28°C on Bacto-agar and glucose (BG) medium. *Pseudomonas syringae* strain DC3000 Δ*hopQ1-1* (Wei et al., 2007) was cultured at 28°C on Luria-Marine (LM) agar medium plate.

### Plasmid constructions

The nucleic acid sequence and amino acid sequence of RipAW in *R. solanacearum* strain GMI1000 were obtained from NCBI^1^ and analyzed using SMART^2^. All the DNA fragments used to generate overexpression and silencing constructs were amplified by PCR from cDNA of *N. benthamiana* or genomic DNA of *R. solanacearum*. RipAW and its derivatives were cloned into pGWB505 or pCambia2300. The RipAW^C177A^ mutant was generated by site-directed mutagenesis using corresponding primers. Fragments for virus-induced gene silencing (VIGS) were cloned into vector pTRV2. All the primers used for this experiment were listed in Supplementary Table S1.

### *Agrobacterium*-mediated transient expression and cell death assays

The *A. tumefaciens* cells harboring the corresponding plasmids were suspended in an infiltration buffer containing 150 mM acetosyringone, 10 mM MgCl_2_ and 10 mM MES (pH 5.6), and adjusted to an OD_600_ of 0.5. The inoculum preparations were incubated at 28°C for 2 h. The inoculum preparations were then infiltrated into leaves of five-week-old *N. benthamiana* using a needless syringe. The infiltrated leaves were photographed with a digital camera at 48 or 72 h post infiltration to visualize cell death triggered by effectors.

### Pathogen inoculations

*Ralstonia solanacearum* strain CQPS-1 and *P. syringae* strain DC3000 Δ*hopQ1-1* were grown in BG and LM liquid medium, respectively, at 28°C overnight. Bacterial cells were collected by centrifugation, washed with sterile water and adjusted to a final OD_600_ of 0.001 for CQPS-1 and 0.00002 for DC3000 Δ*hopQ1-1*. RipAW or its derivatives including RipAW^C177A^, RipAW^N1-90^, RipAW^NEL^, RipAW^C322-448^, RipAW ΔN and RipAW ΔC was transiently expressed in *N. benthamiana* leaves. LTI6b, a small intrinsic protein not related to plant immunity (Martinière et al., 2011), was commonly used as the control in plant immunity study (Lee et al., 2020). Therefore, LTI6b was transiently expressed as a control. The leaves were inoculated with CQPS-1 or DC3000 Δ*hopQ1-1* 48 h post-infiltration, and the bacterial titers were measured at 2–3 days post-inoculation (dpi).

### qRT-PCR

Total RNA was extracted using the RNAsimple Total RNA Extraction Kit (TIANGEN), and quantitative expression of the defense-related genes *NbHIN1* and *NbPR1a* was determined by qRT-PCR with SYBR Green PCR Master Mix as previously described (An et al., 2022). *NbEF1α* was selected as an internal control. The primers used for amplification were listed in Supplementary Table S1.

### Western blot analysis

Total soluble proteins were extracted from *N. benthamiana* leaves expressing RipAW, RipE1, INF1, NahG or RipAW’s derivatives. Proteins were separated by SDS-PAGE and transferred onto PVDF membranes (Thermo Fisher) which were then blocked using 5% defatted milk in TBST buffer (1× TBS containing 0.05% Tween 20) for 60 min at room temperature. The membranes were incubated with anti-FLAG (1:8000, Sigma), anti-HA (1:10000, Abmart) or anti-GFP (1:8000, ABclonal) at room temperature for 1 h and then with a horseradish peroxidase-conjugated secondary antibody (1:10000, Sigma) for another 45 min. Signals were detected and photographed using the SuperSignal West Pico Chemiluminescent substrate (Thermo Scientific) and the Bio-Rad ChemiDoc Touch Imaging System.

### Ion leakage assay

The extent of plant cell death can be quantified by electrolyte leakage according to Sang et al. (2020). After 36 h of transient expression of effectors in *N. benthamiana* leaves, leaf discs were collected with a hole punch (1.2 cm in diameter) and immersed in 5 mL of sterile water. After 1 h of gentle shaking at room temperature, the ion conductivity of the water measured using a conductivity meter Waterproof Pocket Tester (Oakton). Three replicates were set up in parallel for each group of experiments.

### VIGS assay

VIGS assay using TRV (tobacco rattle virus) vectors in *N. benthamiana* was carried out according to Senthil-Kumar and Mysore (2014). For TRV-mediated gene silencing, *A. tumefaciens* cultures expressing *TRV2::NbSGT1* and those expressing *TRV1* were mixed at 1:1 ratio to a final OD_600_ of 1.0, and infiltrated into the first two true leaves of two-week-old *N. benthamiana*. After being covered with plastic film to keep high humidity for 2 days, plants were cultured under normal conditions. *TRV2::GFP* was generated as control plants. Three-week-old plants after VIGS were used for corresponding assays.

### Statistical analysis

All experiments were repeated for at least three times with similar results. Statistical analysis was performed using Student’s *t*-test along with analysis of variance to discriminate statistical differences between treatments.

## Results

### RipAW^C177A^ does not induce cell death but triggers plant immunity

**Figure 1:**
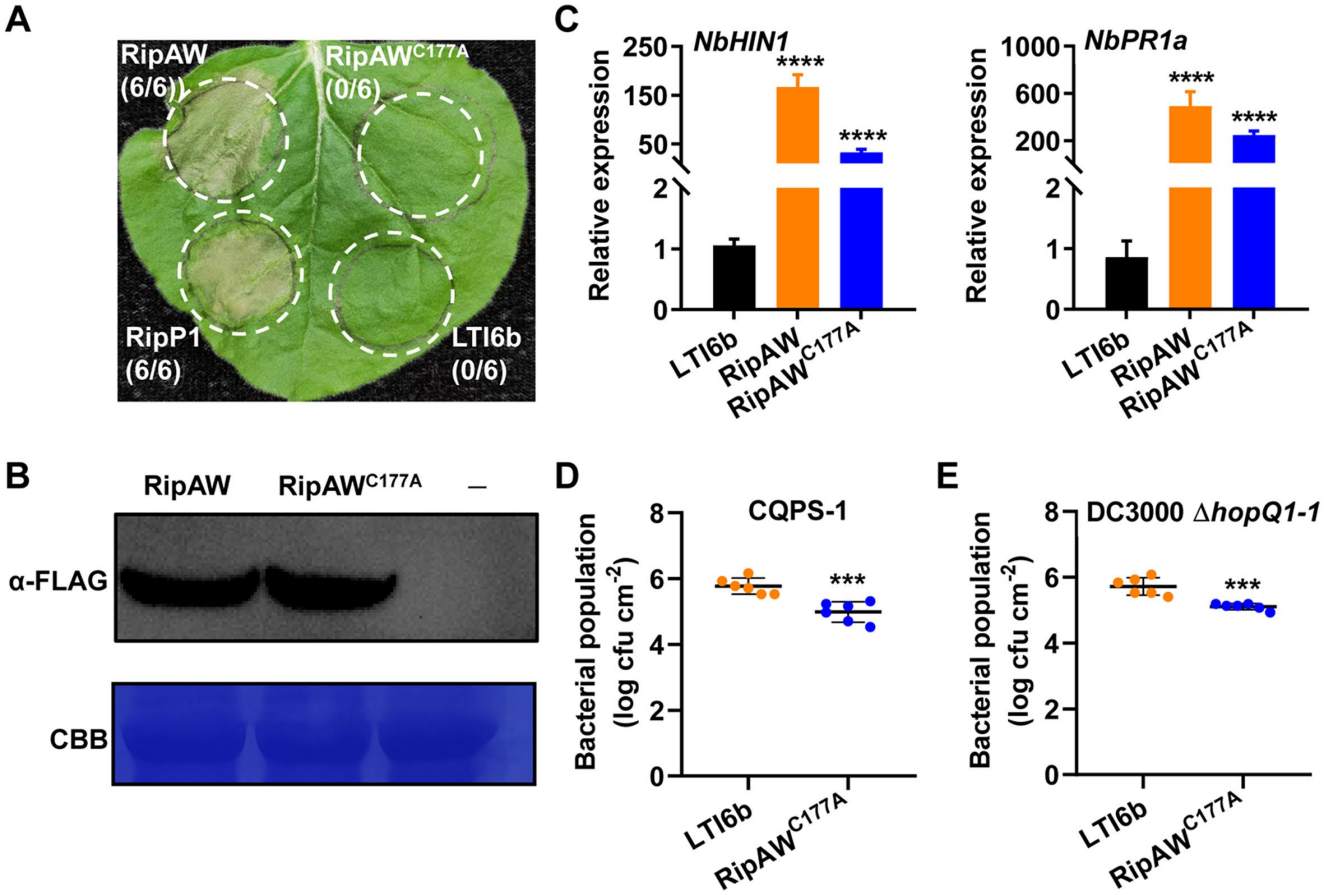
Plant immunity triggered by RipAW^C177A^ in *Nicotiana benthamiana*. **(A)** The RipAW^C177A^ mutant failed to induce cell death in *N. benthamiana*. RipAW and RipAW^C177A^ were expressed in the same leaf of *N. benthamiana*. RipP1 and LTI6b were the positive control and negative control, respectively. Photographs were taken 2 days post inoculation (dpi). Circles indicate the infiltrated areas. The fractions in brackets represent the number of leaves displaying HR over the total number of infiltrated leaves. **(B)** Detection of RipAW and RipAW^C177A^ protein expression by western blot using anti-FLAG antibody. Total protein was extracted from the leaves 2 days after agroinfiltration and detected by western blot. The blot was stained with Coomassie brilliant blue (CBB) to confirm equal loading. **(C)** Expression of defense-related genes *NbHIN1* and *NbPR1a* in *N. benthamiana* leaves expressing LTI6b (control), RipAW, or RipAW^C177A^ by qRT–PCR. **(D,E)** Growth of *Ralstonia solanacearum* CQPS-1 **(D)** and *Pseudomonas syringae* DC3000 Δ*hopQ1-1* **(E)** in *N. benthamiana* leaves expressing RipAW^C177A^ and LTI6b. RipAW^C177A^ and LTI6b were agroinfiltrated into *N. benthamiana* leaves. 48 h later, CQPS-1 or DC3000 Δ*hopQ1-1* was infiltrated into the same areas. The bacterial population was determined at 2 dpi for CQPS-1 and 3 dpi for DC3000 Δ*hopQ1-1*. Values are means ± standard errors (SEs) from six biological replicates. *** and **** indicate significant differences at *p* ≤ 0.001 and *p* ≤ 0.0001, respectively.

RipAW is a new member of the NEL family (Nakano et al., 2017), and it has been reported that its E3 ligase activity is required for RipAW-induced cell death in *N. benthamiana* (Niu et al., 2022). Here, RipAW in *R. solanacearum* strain GMI1000 induced cell death in *N. benthamiana*, but its E3 ligase mutant RipAW^C177A^ could not (Figure 1A). Western blot analysis using the anti-FLAG antibody confirmed the expression of RipAW and RipAW^C177A^ proteins (Figure 1B). These results confirmed the essential role of its E3 ligase activity in RipAW-induced cell death. To further define the role of E3 ligase activity in RipAW-triggered immunity, we detected the expression of two famous defense-related genes *NbHIN1* and *NbPR1a* (Delaney et al., 1994; Gopalan et al., 1996) in *N. benthamiana* leaves expressing RipAW or RipAW^C177A^. Compared to the control (LTI6b), RipAW expression extremely increased the expression level of *NbHIN1* and *NbPR1a* (Figure 1C). Unexpectedly, expressing RipAW^C177A^ also significantly promoted expression of *NbHIN1* and *NbPR1a*, although not as high as RipAW did. In addition, we evaluated whether the overexpression of RipAW^C177A^ affected plant resistance to pathogens. After expression of RipAW^C177A^ in *N. benthamiana*, tobacco leaves were inoculated with *R. solanacearum* strain CQPS-1 or *P. syringae* strain DC3000 Δ*hopQ1-1*. The result showed that RipAW^C177A^ expression significantly restricted the growth of CQPS-1 (Figure 1D) and DC3000 Δ*hopQ1-1* (Figure 1E). These results indicate that the E3 ligase activity of RipAW is not essential for induction of plant immunity.

### The N terminus, C terminus and NEL domain are all required but not sufficient for RipAW to induce cell death

**Figure 2:**
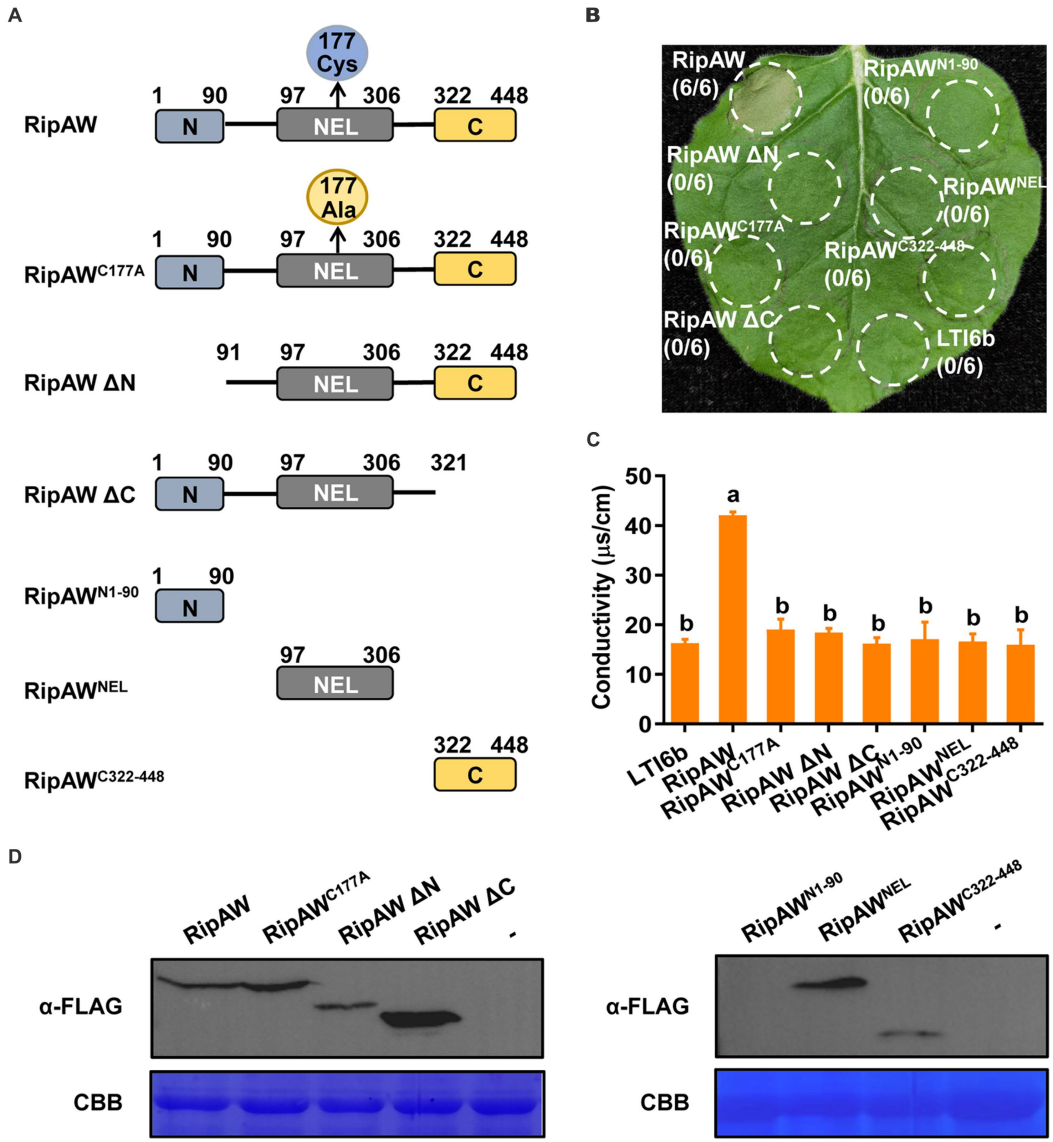
Induction of cell death by RipAW and its mutants. **(A)** Schematic illustration of RipAW and its derivatives RipAW^C177A^ (cysteine at position 177 of RipAW is substituted by alanine), RipAW ΔN, RipAW ΔC, RipAW^N1-90^, RipAW^NEL^ and RipAW^C322-448^. The N-terminus, NEL domain and C-terminus of RipAW are indicated as blue, gray and yellow boxes, respectively. The positions of amino acid residues are indicated by numbers. **(B)** RipAW induces cell death in *N. benthamiana*, but all its derivatives do not. RipAW and its derivatives were expressed in the same leaf of *N. benthamiana*. LTI6b was the negative control. Photographs were taken at 3 dpi. Circles indicate the infiltrated areas. The fractions in brackets represent the number of leaves displaying HR over the total number of infiltrated leaves. **(C)** Cell death in **(B)** was evaluated by the degree of ion leakage. The degree of ion leakage from the leaf discs was measured 44 h after agroinfiltration using a conductivity meter. Values are means ± SEs from three biological replicates. Different letters indicate significant differences at *p* ≤ 0.01. **(D)** Detection of RipAW and its derivatives with western blot using anti-FLAG antibody. Total protein was extracted from the leaves 2 days after agroinfiltration. The blot was stained with Coomassie brilliant blue (CBB) to confirm equal loading.

Except the NEL domain, Niu et al. (2022) reported that the N terminus of RipAW is also required for induction of cell death in *N. benthamiana*. To further determine the functional domains in RipAW, we constructed a series of truncated mutants of RipAW. The NEL domain of RipAW is located at amino acids 97–306 (Nakano et al., 2017). We generated mutants including RipAW^N1-90^, RipAW^NEL^, RipAW^C322-448^, N-terminal and C-terminal deletion mutants of RipAW, namely RipAW ΔN and RipAW ΔC, which deleted the N terminus and C terminus of RipAW, respectively (Figure 2A). The above derivatives, as well as wild-type RipAW and RipAW^C177A^, were expressed in the leaves of *N. benthamiana*. We found that, except for the full-length RipAW which induced strong cell death, none of its derivatives could induce cell death in *N. benthamiana* at 3 dpi (Figure 2B). Electrolyte leakage measurement showed that only full-length RipAW induced higher ion leakage, but its derivatives did not (Figure 2C), confirming the cell death phenotypes. Western blot analysis confirmed the expression of RipAW and its derivatives in *N. benthamiana*, except RipAW^N1-90^ which was too small to be detected (Figure 1D). These results indicate that not only the N terminus, the NEL domain but also the C terminus is required for RipAW-induced cell death.

### RipAW derivatives deleting the NEL domain can still trigger immunity

**Figure 3:**
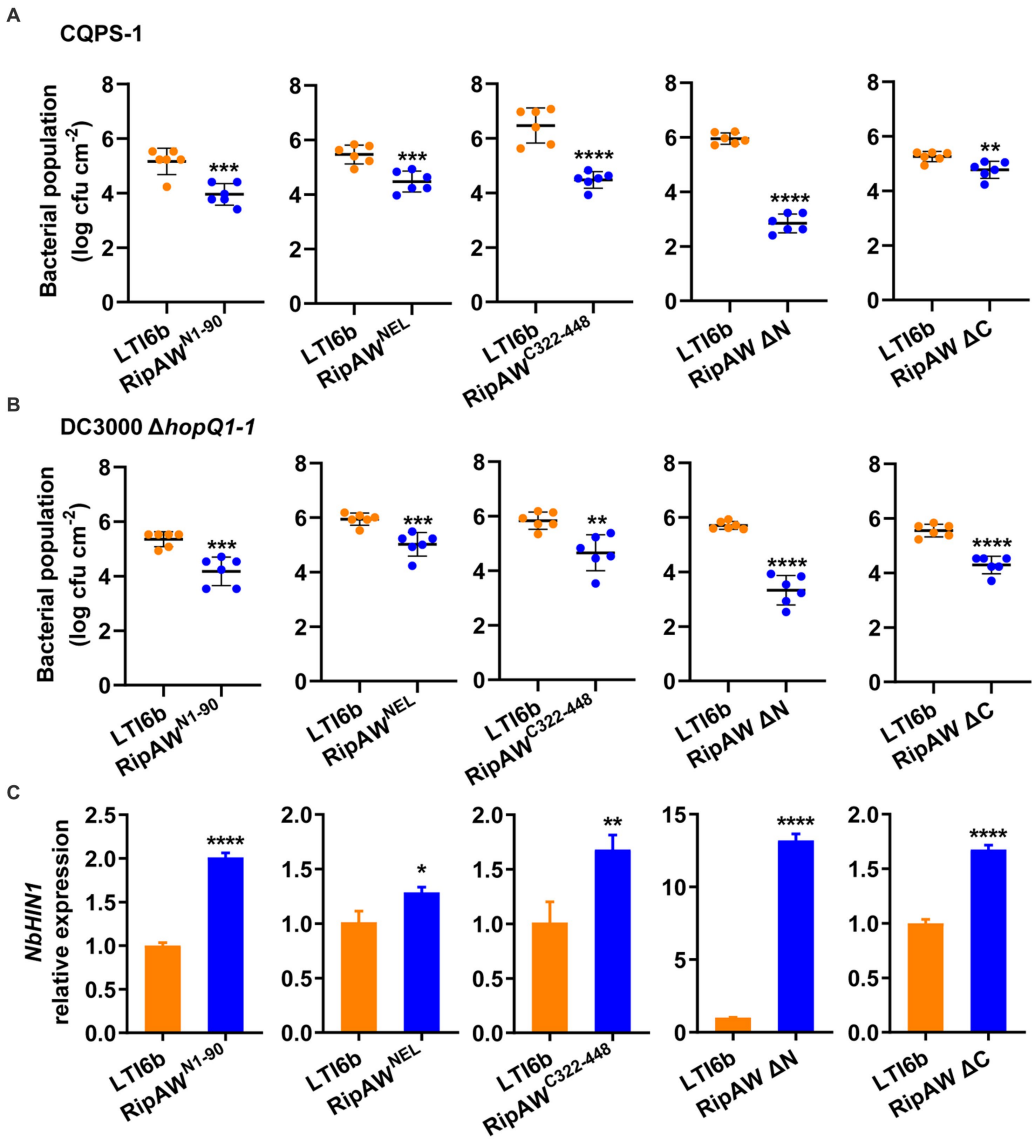
Plant immunity triggered by RipAW mutants. **(A,B)** Bacterial population of *R. solanacearum* CQPS-1 **(A)** and *P. syringae* DC3000 Δ*hopQ1-1* **(B)** in *N. benthamiana* leaves expressing corresponding RipAW derivatives. Agrobacterial cells harboring RipAW mutants or LTI6b were infiltrated into *N. benthamiana* leaves. 48 h later, CQPS-1 or DC3000 Δ*hopQ1-1* was infiltrated into the same areas. The bacterial population was determined at 2 dpi for CQPS-1 and 3 dpi for DC3000 Δ*hopQ1-1*. **(C)** *NbHIN1* expression in *N. benthamiana* leaves expressing LTI6b (control) or RipAW’s derivatives by qRT–PCR. Values are means ± SEs from three biological replicates. *, **, ***, and **** indicate significant differences at *p* ≤ 0.05, *p* ≤ 0.01, *p* ≤ 0.001 and *p* ≤ 0.0001, respectively.

The E3 ligase mutant RipAW^C177A^ still triggered plant immunity (Figure 1), indicating that some other regions also contribute to RipAW-triggered immunity. We expressed RipAW derivatives individually in *N. benthamiana*, and investigated their effects on plant resistance to CQPS-1 and DC3000 Δ*hopQ1-1*. Results showed that, compared with the control, expression of RipAW^N1-90^, RipAW^NEL^, RipAW^C322-448^, RipAW ΔN and RipAW ΔC all significantly suppressed the colonization of both CQPS-1 (Figure 3A) and DC3000 Δ*hopQ1-1* (Figure 3B) in *N. benthamiana*. These results indicate that the N- and C-terminal regions of RipAW can also trigger plant immunity. Meanwhile, we examined the expression of the plant immune-related gene *NbHIN1* and found that it was up-regulated significantly by these RipAW derivatives (Figure 3C). This result further confirmed that RipAW-induced plant immunity is not completely dependent on the E3 ubiquitin ligase activity of RipAW.

### Silencing SGT1 impairs RipAW- and RipAW^C177A^-triggered immunity

**Figure 4:**
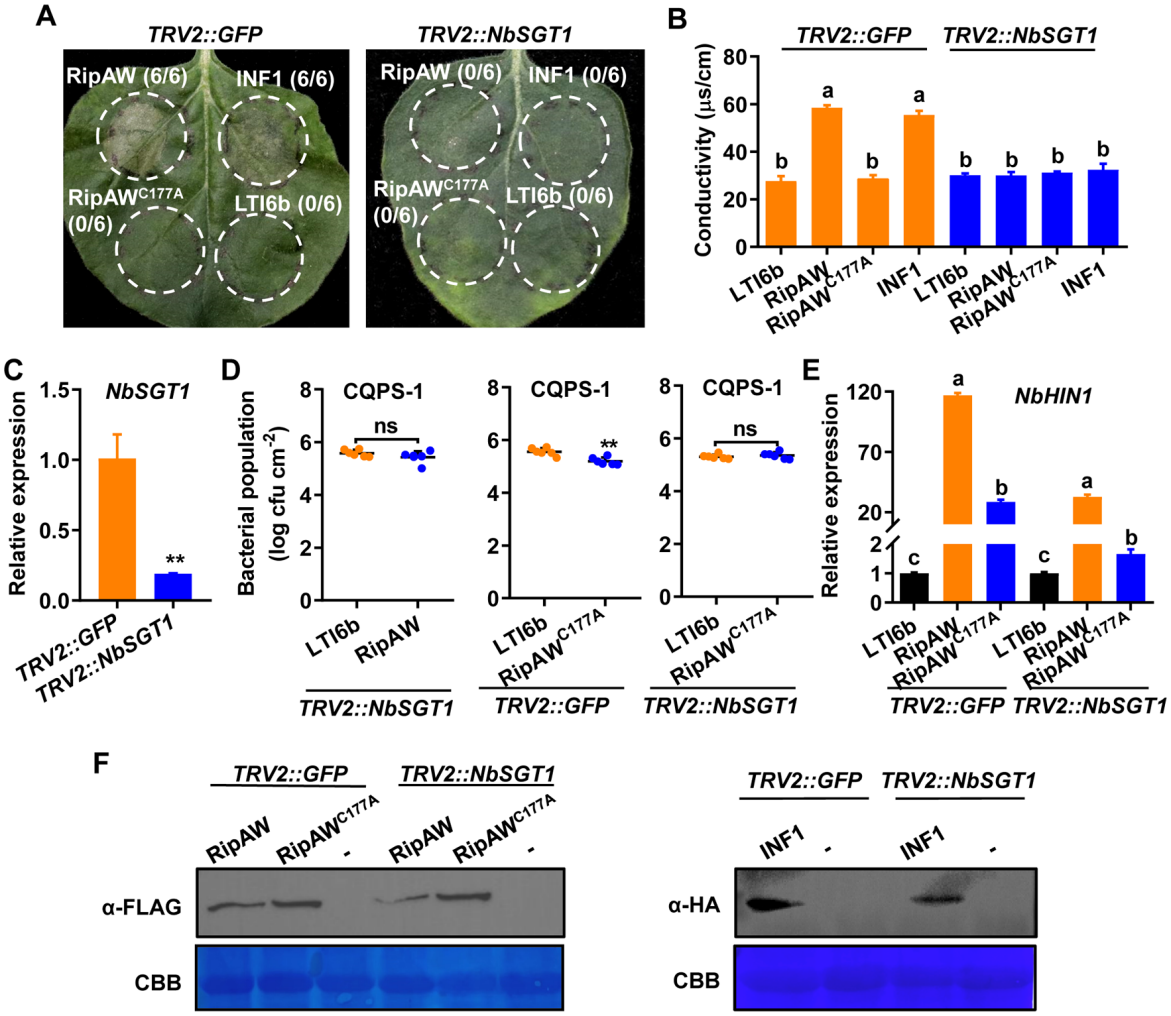
Silencing *NbSGT1* impairs defense triggered by RipAW and RipAW^C177A^. **(A)** Both RipAW and RipAW^C177A^ cannot induce cell death in *NbSGT1*-silenced *N. benthamiana* leaves. Photographs were taken at 3 days after infiltration. Circles indicate the infiltrated areas. The fractions in brackets represent the number of leaves displaying HR over the total number of infiltrated leaves. **(B)** Cell death in **(A)** was evaluated by the degree of ion leakage. The degree of ion leakage from the leaf discs was measured 2 days after agroinfiltration using a conductivity meter. **(C)** *NbSGT1* expression in leaves of *TRV2::NbSGT1* and *TRV2::GFP N. benthamiana*. **(D)** Growth of *R. solanacearum* CQPS-1 in *TRV2::NbSGT1* and *TRV2::GFP N. benthamiana* leaves expressing RipAW and RipAW^C177A^. LTI6b, RipAW and RipAW^C177A^ were agroinfiltrated into *TRV2::NbSGT1* and *TRV2::GFP N. benthamiana* leaves, respectively. 48 h later, CQPS-1 was infiltrated into the same areas. The bacterial population was determined at 2 dpi. **(E)** *NbHIN1* expression in *TRV2::NbSGT1* and *TRV2::GFP N. benthamiana* leaves expressing LTI6b (control), RipAW or RipAW^C177A^ by qRT–PCR. Values in **(B–E)** are means ± SEs from at least three biological replicates. Different small letters in **(B,E)** and ** in panels **(C,D)** indicate significant differences at *p* ≤ 0.001. ns indicates no significant difference. **(F)** Detection of RipAW, RipAW^C177A^ and INF1 in *TRV2::NbSGT1* and *TRV2::GFP N. benthamiana* by western blot using anti-FLAG or anti-HA antibody. Total protein was extracted from the leaves 2 days after agroinfiltration. The blot was stained with Coomassie brilliant blue (CBB) to confirm equal loading.

SGT1 is a major ETI regulator (Azevedo et al., 2002; Kadota et al., 2010), and RipAW-induced cell death has been reported to be SGT1-dependent (Niu et al., 2022). We wonder whether RipAW-triggered plant immunity is also SGT1-dependent. We silenced SGT1 in *N. benthamiana* by VIGS and tested RipAW- and RipAW^C177A^-induced immune responses in the *NbSGT1*-silenced plants. Results showed that, similar to INF1 whose cell death induction is dependent on SGT1 (Bos et al., 2006), RipAW could not induce cell death in the *NbSGT1-*silenced *N. benthamiana* (Figure 4A). The ubiquitin E3 ligase mutant RipAW^C177A^ could not induce cell death in both control and the *NbSGT1*-silenced plants. The ion leakage results confirmed the results of cell death (Figure 1B). qRT-PCR analysis showed that *NbSGT1* expression in *TRV2::NbSGT1 N. benthamiana* was only 18.81% of that in *TRV2::GFP* plants (Figure 4C), indicating that *NbSGT1* was successfully silenced. Expression of RipAW^C177A^ significantly decreased CQPS-1’s population in *TRV2::GFP* plants, but this effect was abolished in *NbSGT1*-silenced plants (Figure 4D). Expression of RipAW also failed to promote plant resistance to CQPS-1 in *NbSGT1*-silenced plants (Figure 4D). These results indicate that not only RipAW-induced cell death but also RipAW-triggered immunity is dependent on NbSGT1. We further analyzed the expression of defense-related gene *NbHIN1* and found that both RipAW- and RipAW^C177A^-enhanced *NbHIN1* expression was significantly impaired in the *NbSGT1*-silenced plants compared with that in control (*TRV2::GFP*) plants (Figure 4E), confirming the important role of NbSGT1 in RipAW and RipAW^C177A^-triggered plant immunity. Western blot analysis showed the successful expression of RipAW, RipAW^C177A^ and INF1 proteins (Figure 4F).

### RipAW- and RipAW^C177A^-triggered immunity is independent of EDS1 and helper NLRs

**Figure 5:**
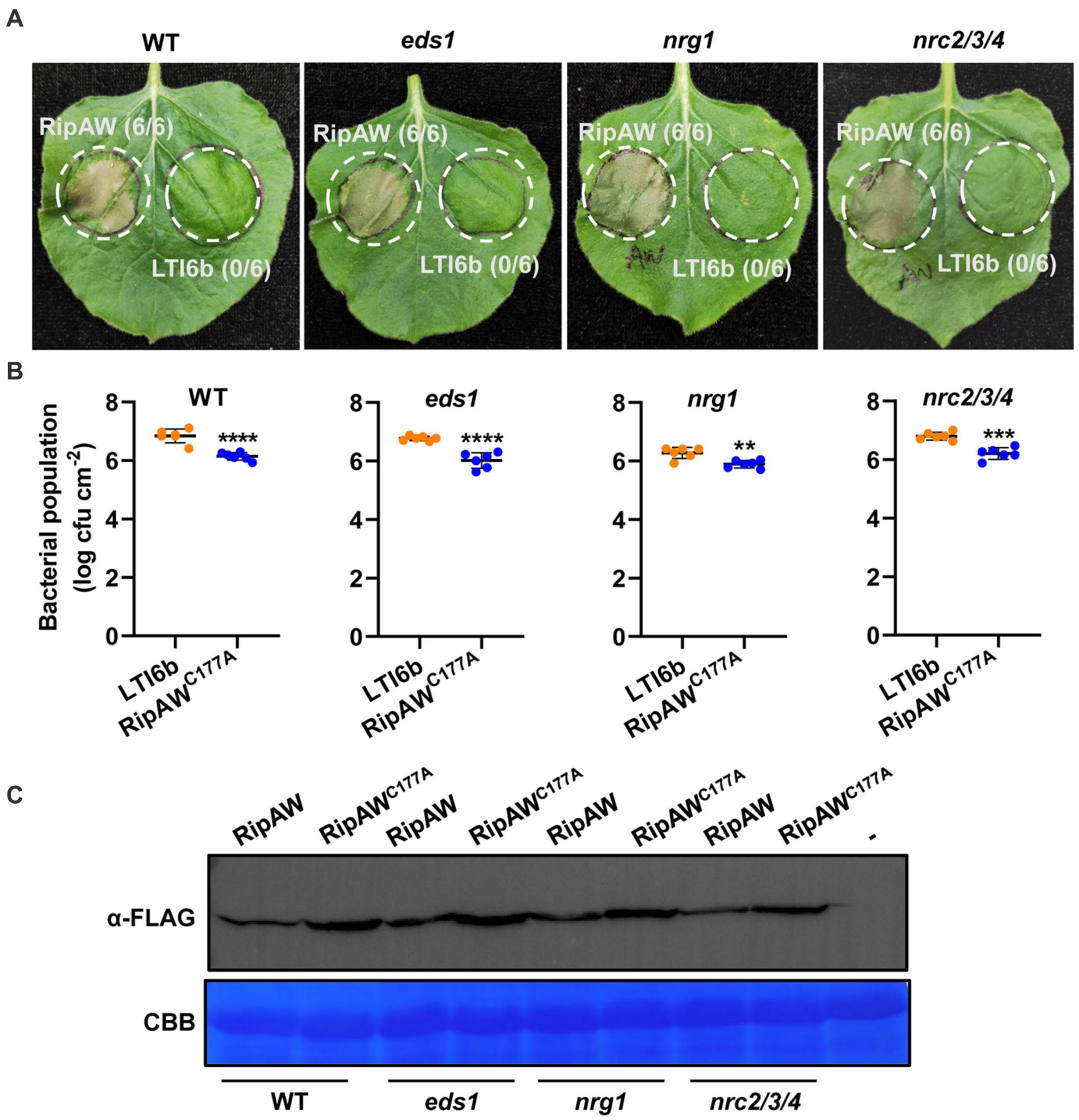
RipAW^C177A^-triggered immunity in *eds1* and mutants of Helper NLRs. **(A)** RipAW-induced cell death in wild-type (WT) *N. benthamiana* and *eds1*, *nrg1*, *nrc2/3/4* mutants. RipAW and LTI6b (control) were expressed in the same leaf of wild-type *N. benthamiana*, *eds1*, *nrg1* and *nrc2/3/4* mutants, respectively. Photographs were taken at 3 dpi. Circles indicate the infiltrated areas. The fractions in brackets represent the number of leaves displaying HR over the total number of infiltrated leaves. **(B)** Growth of *R. solanacearum* CQPS-1 in WT and mutant leaves expressing LTI6b or RipAW^C177A^. LTI6b and RipAW^C177A^ were agroinfiltrated into WT and mutant leaves. 48 h later, CQPS-1 was infiltrated into the same areas. The bacterial population was determined at 2 dpi. Values are means ± SEs from six biological replicates. ** and *** indicate significant differences at *p* ≤ 0.01 and *p* ≤ 0.001, respectively. **(C)** Detection of RipAW and RipAW^C177A^ in WT and mutants by western blot using anti-FLAG antibody. Total protein was extracted from the leaves 2 days after agroinfiltration and subjected to western blot analysis. The blot was stained with Coomassie brilliant blue (CBB) to confirm equal loading.

Although most NLRs require SGT1 to function, EDS1 and helper NLR NRG1 also play critical roles in some TIR-NLRs (Wiermer et al., 2005; Bos et al., 2006; Schultink et al., 2017; Ngou et al., 2022b) and a specific subset of CC-NLRs requires another class of helper NLRs termed NRC proteins (Wu et al., 2017). To determine whether RipAW-triggered plant immunity depends on these important ETI components, we expressed *RipAW*/*RipAW^C177A^* in *eds1*, *nrg1* and *nrc2/3/4* mutants, respectively. We found that RipAW-induced cell death was not affected in *eds1*, *nrg1* and *nrc2/3/4* mutants (Figure 5A). Bacterial population assay further showed that RipAW^C177A^ maintained its function to restrict CQPS-1 growth in *eds1*, *nrg1* and *nrc2/3/4* mutants (Figure 5B). RipAW and RipAW^C177A^ proteins were successfully expressed in both WT and tested mutants (Figure 5C). These results indicate that RipAW- and RipAW^C177A^-triggered plant immunity do not depend on EDS1 and helper NLRs NRG1 and NRCs.

### NahG expression fails to block RipAW-induced cell death and immunity

**Figure 6:**
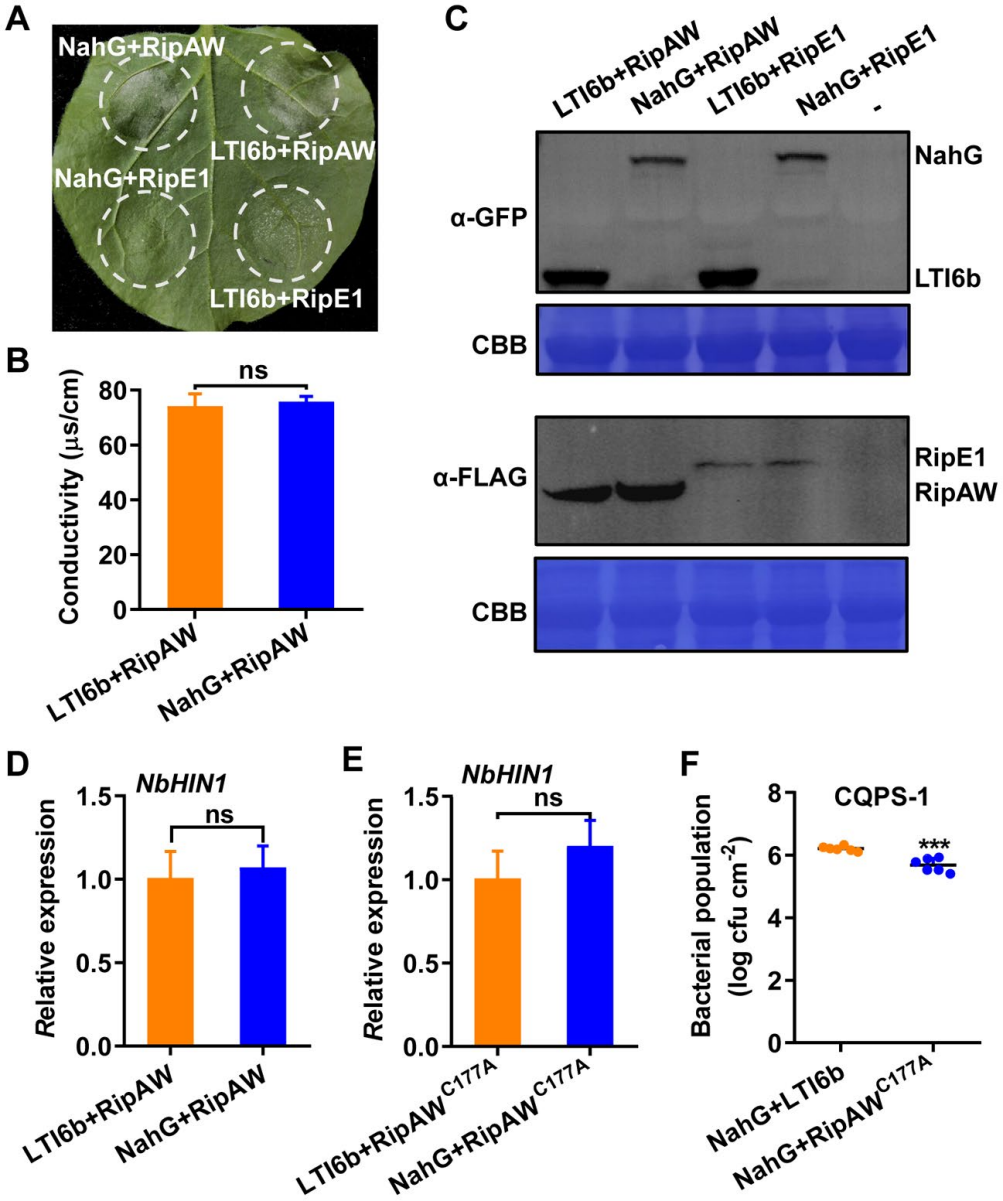
RipAW^C177A^-triggered immunity is independent of SA pathway. **(A)** RipAW induces cell death when co-expressed with NahG. RipAW and RipE1 were co-expressed with LTI6b (control) or NahG in the same leaf. Photographs were taken at 3 dpi. Circles indicate the infiltrated areas. The fractions in brackets represent the number of leaves displaying HR over the total number of infiltrated leaves. **(B)** Cell death in **(A)** was quantified by the degree of ion leakage. The degree of ion leakage from the leaf discs was measured 2 days after agroinfiltration using a conductivity meter. **(C)** Detection of proteins in *N. benthamiana* by western blot using anti-FLAG (for RipAW and RipE1) or anti-GFP (for LTI6 and NahG) antibody. Total protein was extracted from the leaves 2 days after agroinfiltration. The blot was stained with Coomassie brilliant blue (CBB) to confirm equal loading. **(D)** *NbHIN1* expression in *N. benthamiana* leaves co-expressing LTI6b (control) or NahG with RipAW by qRT–PCR. **(E)** *NbHIN1* expression in *N. benthamiana* leaves co-expressing LTI6b (control) or NahG with RipAW^C177A^ by qRT–PCR. **(F)** Growth of *R. solanacearum* CQPS-1 in *N. benthamiana* leaves co-expressing NahG with LTI6b (control) or RipAW^C177A^. LTI6b and RipAW^C177A^ were agroinfiltrated into *NahG*-expressed *N. benthamiana* leaves, respectively. 24 h later, CQPS-1 was infiltrated into the same leaf areas. The bacterial population was determined at 2 dpi. Values are means ± SEs from six biological replicates. *** indicates significant differences at *p* ≤ 0.001 and ns indicates no significant difference.

SA plays a vital role in the activation of immune responses (Zhou and Zhang, 2020). RipE1, another T3E in *R. solanacearum*, has been reported to trigger plant immunity by regulating SA-dependent responses (Sang et al., 2020). Here, we co-expressed *RipAW* or *RipE1* (as a positive control) with *NahG* which blocks SA accumulation in plant (Delaney et al., 1994). Results showed that co-expression of NahG obviously impaired RipE1-induced cell death but did not affect RipAW-induced cell death (Figure 6A) which was further confirmed by the ion leakage result (Figure 6B). NahG, RipAW and RipE1 were all expressed successfully in our experiment (Figure 6C). These results indicate that, different with RipE1, RipAW-triggered plant immunity is probably independent of SA signaling pathway. To confirm this, we investigated the effect of NahG co-expression on RipAW and RipAW^C177A^-induced *NbHIN1* gene expression. We found that RipAW and RipAW^C177A^-induced *NbHIN1* gene expression was not influenced by NahG (Figures 6D,E). In addition, NahG co-expression also did not affect RipAW^C177A^-restricted CQPS-1 growth in *N. benthamiana* (Figure 6F). These results confirm that RipAW-triggered cell death and immunity is independent on SA pathway.

## Discussion

As one of the most destructive bacterial phytopathogens, *R. solanacearum* is endowed with an exceptionally large repertoire of effectors that functions in its interaction with plants (Mansfield et al., 2012; Coll and Valls, 2013). Since ubiquitination is important in the regulation of many cellular processes of plants (Rytkonen and Holden, 2007), it is not surprising that some phytopathogens secrete effectors to interfere extensively with ubiquitination pathways. For example, *P. syringae* type III effector AvrPtoB depends on its E3 ligase activity to degrade NPR1 (Non-expresser of PR genes 1) and hence represses SA-dependent immune responses (Chen et al., 2017). NEL is a novel class of E3 ligase which is distinct from typical RING- and HECT-types (Ashida and Sasakawa, 2016). Type III effector RipAW in *R. solanacearum* strain RS1000 was found to have a NEL domain and suppress plant PTI responses in a E3 ligase activity-dependent manner (Nakano et al., 2017). Niu et al. (2022) further demonstrated that RipAW in *R. solanacearum* strain GMI1000 induced cell death in *N. benthamiana* which requires its E3 ligase activity. These previous studies demonstrate the importance of the E3 ligase activity for the function of RipAW in the plant-*R. solanacearum* interactions. Here, we further defined the role of E3 ligase activity in RipAW-triggered plant immunity, providing new insights into the functional mechanism of RipAW in *R. solanacearum*.

RipAW^C177A^, the E3 ligase mutant of RipAW, lost the ability to induce cell death but retained that to trigger plant immunity in *N. benthamiana* (Figure 1), indicating that the E3 ligase is not essential for RipAW-triggered immunity. All truncated mutants of RipAW, including RipAW^N1-90^, RipAW^NEL^, RipAW^C322-448^, RipAW ΔN and RipAW ΔC, showed the ability to trigger plant immunity without inducing cell death (Figures 2, 3), confirming that the NEL contributes to but is not essential for RipAW-triggered immunity. Niu et al. (2022) demonstrated that both the N-terminal region and NEL domain are required for RipAW-induced cell death. Here, we confirmed Niu et al. (2022)‘s finding and further provided evidence that the C-terminal region is also required for RipAW-induced cell death in *N. benthamiana*. Although being required, none of them alone is sufficient to trigger cell death in *N. benthamiana*.

Early in 2000, researchers found that cell death associated with HR to CaMV was uncoupled genetically from plant resistance (Greenberg et al., 2000). Several following studies also showed that ETI-induced cell death was sometimes segregated from plant immunity (Glocova et al., 2005; Menna et al., 2015; Pitsili et al., 2020; Yang et al., 2022). Recently, the *Xanthomonas* effector XopQ-triggered immune responses were found to be separated from ETI-induced cell death in *N. benthamiana* (Prautsch et al., 2023). In the present study, RipAW^C177A^ and RipAW’s derivatives triggered plant immunity to restrict bacterial infection without inducing ETI-triggered cell death, which is consistent with these previous studies. These results indicate that different ETI outputs are not always coupled together and plant ETI responses may be triggered without inducing cell death. Therefore, except cell death, it would be better to simultaneously consider some other immune responses as outputs when screening for potential effectors in pathogens that are recognized by plants.

SGT1 acts as a molecular chaperone for NLR proteins and has been proved as a key signaling component in ETI triggered by several effectors, such as *Phytophthora infestans* RXLR effector AVR3a (Bos et al., 2006), *R. solanacearum* effector RipB (Nakano and Mukaihara, 2019) and RipE1 (Jeon et al., 2020). RipAW-induced cell death also requires SGT1 (Niu et al., 2022). Here, using *NbSGT1*-silenced plants, we further showed that RipAW- and RipAWC^177A^-triggered plant immunity which is likely uncoupled from cell death is also dependent on SGT1 (Figure 4). Except SGT1, EDS1 and some helper NLRs such as NRG1 and NRCs are also well-known downstream regulators in plant ETI (Peart et al., 2005; Wiermer et al., 2005; Schultink et al., 2017; Wu et al., 2017). However, in this study, both RipAW-induced cell death and RipAW^C177A^-induced resistance to bacterial growth retained in *eds1*, *nrg1* and *nrc2/3/4* mutants (Figure 5), suggesting that RipAW-triggered plant immunity is independent of EDS1, NRG1 and NRC2/3/4. NahG blocks SA accumulation in plants (Delaney et al., 1994). RipE1 was found to trigger cell death partially through SA signaling pathway by co-expressing with NahG (Sang et al., 2020). However, NahG did not affect RipAW-induced cell death and RipAW^C177A^-induced resistance, implying that SA signaling pathway is not essential for RipAW and RipAW^C177A^-triggered plant immunity.

In summary, our data demonstrates that the E3 ligase activity is not essential for plant immunity triggered by the NEL effector RipAW in *R. solanacearum*. The N-terminus, C-terminus and NEL domain all contribute to RipAW-triggered immunity in *N. benthamiana*, and they are jointly required for RipAW-induced cell death. RipAW- and RipAW^C177A^-triggered immunity in *N. benthamiana* requires SGT1, but not EDS1, NRG1, NRC proteins or SA pathway. Our findings shed new light on effector-triggered immunity and provide clues for further in-depth study of mechanism underlying RipAW-induced plant immunity.

## Data availability statement

The original contributions presented in the study are included in the article/Supplementary material, further inquiries can be directed to the corresponding authors.

## Author contributions

MZ, PL, and CS conceived and designed the experiments. XO, JC, ZS, and RW performed the experiments. XO, JC, XW, BL, and MZ analyzed the data. PL, CS, and MZ drafted and modified the manuscript. All authors read and approved the manuscript.

## Funding

This research was supported by the National Natural Science Foundation of China (32072399 and 32272641), the Fundamental Research Funds for the Central Universities (GK202201017), and the Program of Fujian Key Laboratory for Monitoring and Integrated Management of Crop Pests (MIMCP-202203).

## Conflict of interest

The authors declare that the research was conducted in the absence of any commercial or financial relationships that could be construed as a potential conflict of interest.

## Publisher’s note

All claims expressed in this article are solely those of the authors and do not necessarily represent those of their affiliated organizations, or those of the publisher, the editors and the reviewers. Any product that may be evaluated in this article, or claim that may be made by its manufacturer, is not guaranteed or endorsed by the publisher.

## Footnotes

^1^https://www.ncbi.nlm.nih.gov/

^2^http://smart.emblheidelberg.de/smart/change_mode.pl
