# Supplementary material for: Ubiquitin E3 ligase activity of Ralstonia solanacearum effector RipAW is not essential for induction of plant defense in Nicotiana benthamiana
Source: Front Microbiol. 2023 May 24;14:1201444. doi: 10.3389/fmicb.2023.1201444 (PMC10244751; doi:10.3389/fmicb.2023.1201444)
Supplement: Supplementary file 1 [file Table_1.DOCX]

**Table S1. Primers used in this study**

| **Primer name** | **Primer sequence 5' to 3'** |
| --- | --- |
| RipAW-F | GCCCCCTTCACCAGTGAATTCATGCGCCCTACCGCCCCT |
| RipAW-R | GACCATGATTACGCCAAGCTTCCAGGTGCGCGATGGCTCG |
| RipAW^C177A^-F | GTGCCGATGCCGACGCCTATGACAACGCCG |
| RipAW^C177A^-R | CGGCGTTGTCATAGGCGTCGGCATCGGCAC |
| p2300-RipAW-F | AGCTTTCGCGAGCTCGGTACCATGGTTTTCCTTGTCCGGAGC |
| p2300-RipAW-R | CTCGAGCTTGCATGCCTGCAGTCACTTGTCATCGTCGTCCTTGTAATCTCCGCCGCGCGCGGCGAC |
| p2300-RipAWΔN-F | AGCTTTCGCGAGCTCGGTACCATGCGCGAGCCCTTGGC |
| p2300-RipAWΔC-F | AGCTTTCGCGAGCTCGGTACCATGGTTTTCCTTGTCCGGAGC |
| p2300-RipAWΔC-R | CTTGCATGCCTGCAGTCACTTGTCATCGTCGTCCTTGTAATCTGACAGCCTGCAGCGC |
| P2300-RipAW^N1-90^-R | CTCGAGCTTGCATGCCTGCAGTCACTTGTCATCGTCGTCCTTGTAATCCGGCGATGACGGTCC |
| P2300-RipAW-^NEL^-F | AGCTTTCGCGAGCTCGGTACCATGTGGGCGCAGGCGTG |
| P2300-RipAW-^NEL^-R | CTCGAGCTTGCATGCCTGCAGTCACTTGTCATCGTCGTCCTTGTAATCTTCGACCGGATGCTGC |
| p2300-RipAW^C322-448^-F | AGCTTTCGCGAGCTCGGTACCATGGAGCAGCGGCCGGA |
| NbEF1α-F | AGAGGCCCTCAGACAAAC |
| NbEF1α-R | TAGGTCCAAAGGTCACAA |
| NbHIN1-F | GGCCGTGGCGGTGATA |
| NbHIN1-R | AGCCTATTATGGCCCTTCCATT |
| NbPR1a-F | CTGAGGGAAGTGGCGATTTC |
| NbPR1a-F | CTCATCGACCCACATCTCAAC |
